# Associations between serum calcium, 25(OH)D level and bone mineral density in older adults

**DOI:** 10.1186/s13018-019-1517-y

**Published:** 2019-12-21

**Authors:** Minbo Liu, Xiaocong Yao, Zhongxin Zhu

**Affiliations:** 1 Department of Orthopaedics, The First People’s Hospital of Xiaoshan District, Hangzhou, 311200 Zhejiang People’s Republic of China; 2 Zhejiang Chinese Medical University, Hangzhou, 310053 Zhejiang People’s Republic of China; 3 Institute of Orthopaedics and Traumatology of Zhejiang Province, No. 548, Binwen Road, Hangzhou, 310053 Zhejiang People’s Republic of China

**Keywords:** Bone mineral density, Calcium, 25(OH)D, NHANES, Cross-sectional study

## Abstract

**Background:**

Calcium and vitamin D play important roles in bone health as essential nutrients. We explored whether serum calcium, 25(OH)D were associated with bone mineral density (BMD) in older adults.

**Methods:**

This cross-sectional study was conducted on a sample of 4595 participants (2281 men and 2314 women) aged ≥ 50 years (from 50 to 85 years, 60.1 ± 8.7 years for men and 62.0 ± 9.7 years for women) from the National Health and Nutrition Examination Survey (NHANES) 2001–2006. The independent variables were serum calcium and 25(OH)D. The dependent variable was lumbar BMD. The other variables were considered potential effect modifiers. We performed weighted multivariate linear regression models and smooth curve fittings to evaluate the associations between them. Subgroup analyses were also performed.

**Results:**

We observed a negative association between serum calcium and lumbar BMD in the fully adjusted model. In the subgroup analyses, this association was no longer significant among males and other race/ethnicity. On the other hand, there was a positive association between serum 25(OH)D and lumbar BMD in the fully adjusted model. In the subgroup analyses, this association did not differ in different age groups, between men and women. However, the association between serum 25(OH)D and lumbar BMD followed a U-shaped curve in Mexican Americans.

**Conclusions:**

This cross-sectional study indicated that serum calcium negatively correlated with lumbar BMD, and serum 25(OH)D positively correlated with lumbar BMD in older adults. However, the association between serum calcium and lumbar BMD in males followed a U-shaped curve.

## Introduction

Calcium and vitamin D play important roles in bone health as essential nutrients. Calcium is the most abundant mineral in the body and is involved in many biological processes [1]. Emphasized by the guidelines, calcium supplementation is commonly taken among the older individuals for the prevention and treatment of osteoporosis [2–4]. However, the association between serum calcium and bone mineral density (BMD) is largely unclear [5, 6]. Moreover, evidence from previous studies indicated that increased serum calcium correlated with an increased risk of other diseases, such as coronary artery disease and stroke [7–10]. Thus, there is a need to understand the potential effects of serum calcium on bone health and balance potential risks against potential benefits.

On the other hand, vitamin D promotes calcium absorption in the gut and resorption in the kidney and stimulates bone formation and remodeling [11]. Vitamin D deficiency is associated with decreased calcium absorption [12]. The relationship of serum 25(OH)D and BMD has been studied extensively over the past decades, but there is still no consensus that higher serum 25(OH)D has positive effects on bone health [13–20].

Worldwide, prevention and treatment of osteoporosis have been two increasingly important public health issues with increasing life expectancy, implying the great need to find more effective ways to reduce the related financial burden [21, 22]. As a clinically relevant measurement, BMD is commonly used for the diagnosis of osteoporosis and osteopenia. Here, we conducted a cross-sectional study to estimate the associations between serum calcium, 25(OH)D level and lumbar BMD in older adults using a large-scale database from National Health and Nutrition Examination Survey (NHANES).

## Methods

### Study population

The NHANES is the only population-based national survey that collected information regarding the health and nutrition in the USA in biennial cycles. Information from NHANES is made available through articles in scientific and technical journals and an extensive series of publications. The survey data are publicly available on the internet for data users and researchers throughout the world. Full details of the design and operation are available at www.cdc.gov/nchs/nhanes/. The data from 2001 to 2006 were combined in this report. Of all the 5382 individuals aged ≥ 50 (from 50 to 85 years) with all serum calcium, 25(OH)D, and lumbar BMD data, 4595 participants (2281 men and 2314 women, 60.1 ± 8.7 years for men and 62.0 ± 9.7 years for women) remained for the final analysis after exclusion of 787 subjects with cancer or malignancy. The ethics review board of the National Center for Health Statistics approved all NHANES protocols and written informed consents were obtained from all participants [23].

### Variables

In this study, the independent variables were serum calcium and 25(OH)D. The dependent variable was lumbar BMD. Serum calcium was determined by Beckman Synchron LX20 (Beckman Coulter, Brea, CA). The vitamin D status has been assessed by serum 25(OH)D level [24]. Serum 25(OH)D was determined by the radioimmunoassay kit (DiaSorin, Stillwater, Minnesota, USA). As the 25(OH)D data from 2003 to 2006 was affected by laboratory drifts, we used the calibrated data (updated November 2010). In the present study, the serum 25(OH)D levels were categorized into three clinically relevant categories: deficient (< 20 ng/mL), intermediate (20 to < 30 ng/mL), and optimal (≥ 30 ng/mL) [25]. Lumbar BMD were measured by DEXA scans.

Continuous variables included age, body mass index (BMI), and poverty to income ratio. Categorical variables included sex, race/ethnicity, educational level, physical activity, smoking behavior, and alcohol consumption. Details of serum calcium, 25(OH)D and lumbar BMD measurement processes and other covariate acquisition processes are available at www.cdc.gov/nchs/nhanes/.

### Statistical analysis

We calculated all estimates accounting for NHANES sample weights. We performed weighted multivariate linear regression models and smooth curve fittings to evaluate the associations between serum calcium, 25(OH)D, and lumbar BMD. The other variables were considered potential effect modifiers. For continuous variables, the weighted linear regression model was used to calculate the differences among different groups. For categorical variables, the weighted chi-square test was used. 0.05 (*P* value) was a significance level. All statistical analyses were carried out with R (http://www.R-project.org, The R Foundation) and EmpowerStats software (http://www.empowerstats.com, X&Y Solutions, Inc., Boston, MA).

## Result

The weighted subject characteristics are shown in Table 1. The database included 2281 men and 2314 women; mean age was 60.1 ± 8.7 years for men and 62.0 ± 9.7 years for women; mean serum calcium was 9.5 ± 0.4 mg/dL for men and 9.5 ± 0.4 mg/dL for women; mean serum 25(OH)D was 23.7 ± 8.4 ng/mL for men and 22.7 ± 9.3 ng/mL for women; mean lumbar BMD was 1.06 ± 0.17 g/cm^2^ for men and 0.99 ± 0.16 g/cm^2^ for women.

**Table 1:** Participant characteristics

Characteristic	Men (*n* = 2281)	Women (*n* = 2314)	*P* value
Age, mean ± SD (years)	60.14 ± 8.68	62.02 ± 9.66	< 0.0001
Race/ethnicity (%)			0.1823
Non-Hispanic White	78.88	77.20	
Non-Hispanic Black	8.96	10.08	
Mexican American	4.57	3.96	
Other race/ethnicity	7.59	8.76	
BMI, mean ± SD (kg/m^2^)	28.73 ± 5.21	29.11 ± 6.90	0.0396
Education (%)			0.0003
Less than high school	19.83	20.75	
High school	23.47	28.09	
More than high school	56.70	51.16	
Income to poverty ratio, mean ± SD	3.39 ± 1.53	3.12 ± 1.58	< 0.0001
Physical activity (MET-based rank) (%)			< 0.0001
Sits, not walk very much	15.16	22.21	
Walk a lot	23.99	28.89	
Climb stairs or hills often	17.50	18.06	
Heavy activity	34.20	25.85	
Not recorded	9.15	4.99	
Smoking behavior (%)			< 0.0001
None	34.76	56.57	
Past	42.60	28.17	
Current	22.64	15.26	
Alcohol consumption (%)			< 0.0001
Non-drinker	32.66	46.92	
Moderate alcohol use	42.60	35.86	
High alcohol use	38.32	17.22	
Serum calcium, mean ± SD (mg/dL)	9.49 ± 0.37	9.54 ± 0.39	< 0.0001
Serum vitamin D, mean ± SD (ng/mL)	23.69 ± 8.35	22.65 ± 9.26	< 0.0001
Lumbar BMD, mean ± SD (g/cm2)	1.06 ± 0.17	0.99 ± 0.16	< 0.0001

Mean ± SD for continuous variables: *P* value was calculated by weighted linear regression model

Percent for categorical variables: *P* value was calculated by weighted chi-square test

*BMI* body mass index, *BMD* bone mineral density

### Association between serum calcium and lumbar BMD

**Fig. 1:**
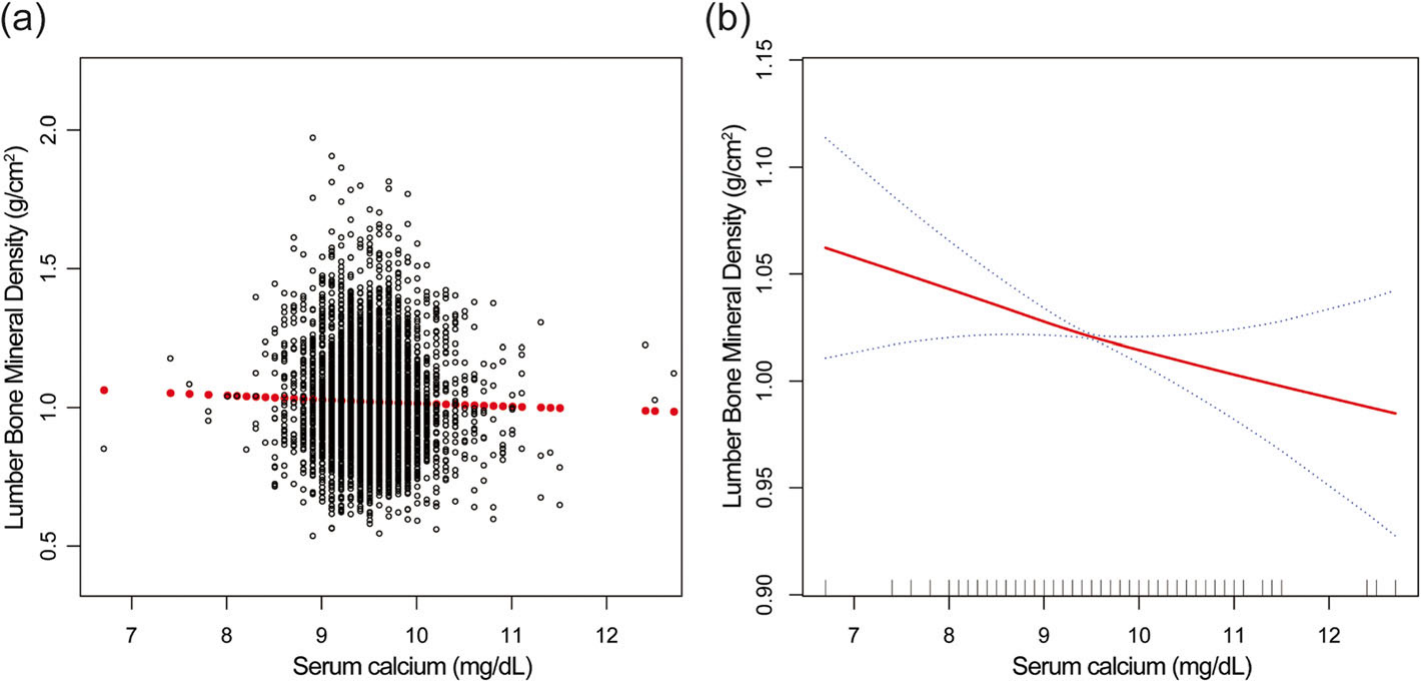
The association between serum calcium and lumbar bone mineral density. **a** Each black point represents a sample. **b** The area between two blue dotted lines is expressed as a 95% CI. Each point shows the magnitude of the serum calcium and is connected to form a continuous line

The effect sizes of association between serum calcium and lumbar BMD are listed in Table 2. In the unadjusted model, we observed a negative association between serum calcium and lumbar BMD. The similar results were found in model 2 (adjustment for age, sex, race/ethnicity) and model 3 (fully adjusted model) [− 0.019 (− 0.031, − 0.007)]. Stratified by tertile of serum calcium, the trend test remained significant between them (*P* = 0.033). We also performed weighted generalized additive models and smooth curve fittings to evaluate the associations between them (Fig. 1).

**Table 2:** Association of serum calcium, 25(OH)D with lumbar bone mineral density

	Model 1 *β* (95% CI)	Model 2 *β* (95% CI)	Model 3 *β* (95% CI)
Serum calcium	− 0.029 (− 0.042, − 0.016)	− 0.028 (− 0.040, − 0.015)	− 0.019 (− 0.031, − 0.007)
Serum calcium (tertile)			
T1	Reference	Reference	Reference
T2	0.017 (− 0.031, − 0.004)	− 0.021 (− 0.034, − 0.008)	− 0.019 (− 0.033, − 0.006)
T3	− 0.023 (− 0.036, − 0.010)	− 0.024 (− 0.036, − 0.012)	− 0.016 (− 0.029, − 0.004)
P for trend	< 0.001	< 0.001	0.033
Serum 25(OH)D	− 0.0002 (− 0.0008, 0.0003)	0.0001 (− 0.0004, 0.0007)	0.0011 (0.0005, 0.0017)
Serum 25(OH)D clinically relevant categories			
< 20 (ng/mL)	Reference	Reference	Reference
20–29.9 (ng/mL)	− 0.001 (− 0.013, 0.010)	0.004 (− 0.007, 0.015)	0.013 (0.002, 0.025)
≥ 30 (ng/mL)	− 0.011 (− 0.024, 0.003)	− 0.004 (− 0.018, 0.009)	0.020 (0.005, 0.034)
*P* for trend	0.155	0.637	0.005

Model 1, no covariates were adjusted

Model 2, age, sex, race/ethnicity were adjusted

Model 3, age, sex, race/ethnicity, body mass index, education, income to poverty ratio, physical activity, smoking behavior, and alcohol consumption were adjusted

In the subgroup analyses stratified by age, sex, or race/ethnicity, the association between serum calcium and lumbar BMD was no longer significant among males and other race/ethnicity (Fig. 3, 4, and 5). For them, lumbar BMD increased with serum calcium up to the turning points. We further examined the threshold effect of serum calcium on lumbar BMD using two-piecewise linear regression models (Table 3).

**Table 3:** Threshold effect analysis of serum calcium and 25(OH)D on lumbar bone mineral density by using piecewise linear regression

	Adjusted *β* (95% CI), *p* value
Males	
Serum calcium < 9.6 (mg/dL)	− 0.028 (− 0.058, 0.003), 0.0724
Serum calcium > 9.6 (mg/dL)	0.025 (− 0.012, 0.061), 0.1915
Other race/ethnicity	
Serum calcium < 9.4 (mg/dL)	− 0.098 (− 0.197, 0.002), 0.0564
Serum calcium > 9.4 (mg/dL)	0.047 (− 0.019, 0.113), 0.1626
Mexican Americans	
Serum 25(OH)D < 23(ng/mL)	− 0.0005 (− 0.0028, 0.0019), 0.6956
Serum 25(OH)D > 23(ng/mL)	0.0020 (− 0.0010, 0.0050), 0.1902

Age, sex, race/ethnicity body mass index, education, income to poverty ratio, physical activity, smoking behavior, and alcohol consumption were adjusted in the model

In the subgroup analysis for males, other race/ethnicity, and Mexican Americans, the model is not adjusted for sex or race/ethnicity, respectively

### Association between serum 25(OH)D and lumbar BMD

**Fig. 2:**
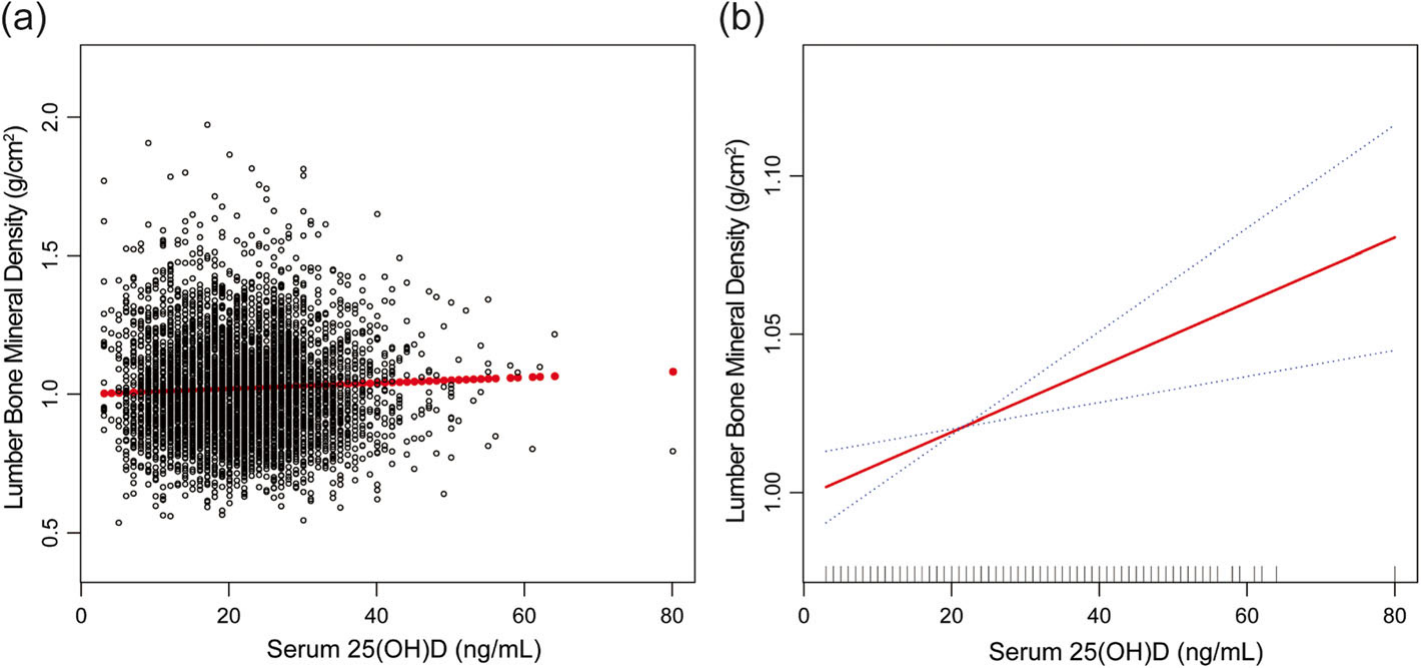
The association between serum 25(OH)D and lumbar bone mineral density. **a** Each black point represents a sample. **b** The area between two blue dotted lines is expressed as a 95% CI. Each point shows the magnitude of the 25(OH)D and is connected to form a continuous line

In model 1 (unadjusted model) and model 2 (adjustment for age, sex, race/ethnicity), the association between serum 25(OH)D and lumbar BMD was not significant, while a positive association between them was observed in the fully adjusted model [0.0011 (0.0005, 0.0017)] (Table 2, Fig. 2). Stratified by clinically relevant categories of serum 25(OH)D, the trend test remained significant between them (*P* = 0.005).

**Fig. 3:**
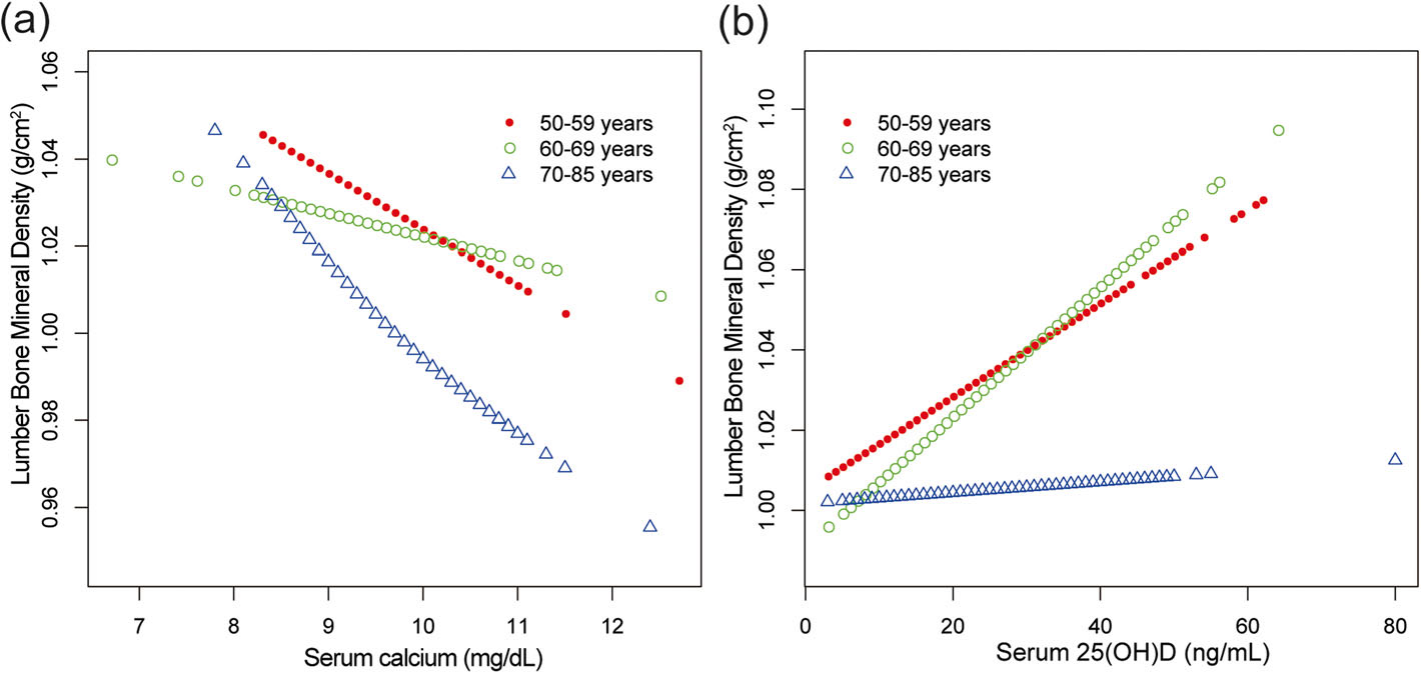
**a**, **b** The associations between serum calcium, serum 25(OH)D and lumbar bone mineral density stratified by age. Age, sex, race/ethnicity, body mass index, education, income to poverty ratio, physical activity, smoking behavior, and alcohol consumption were adjusted

**Fig. 4:**
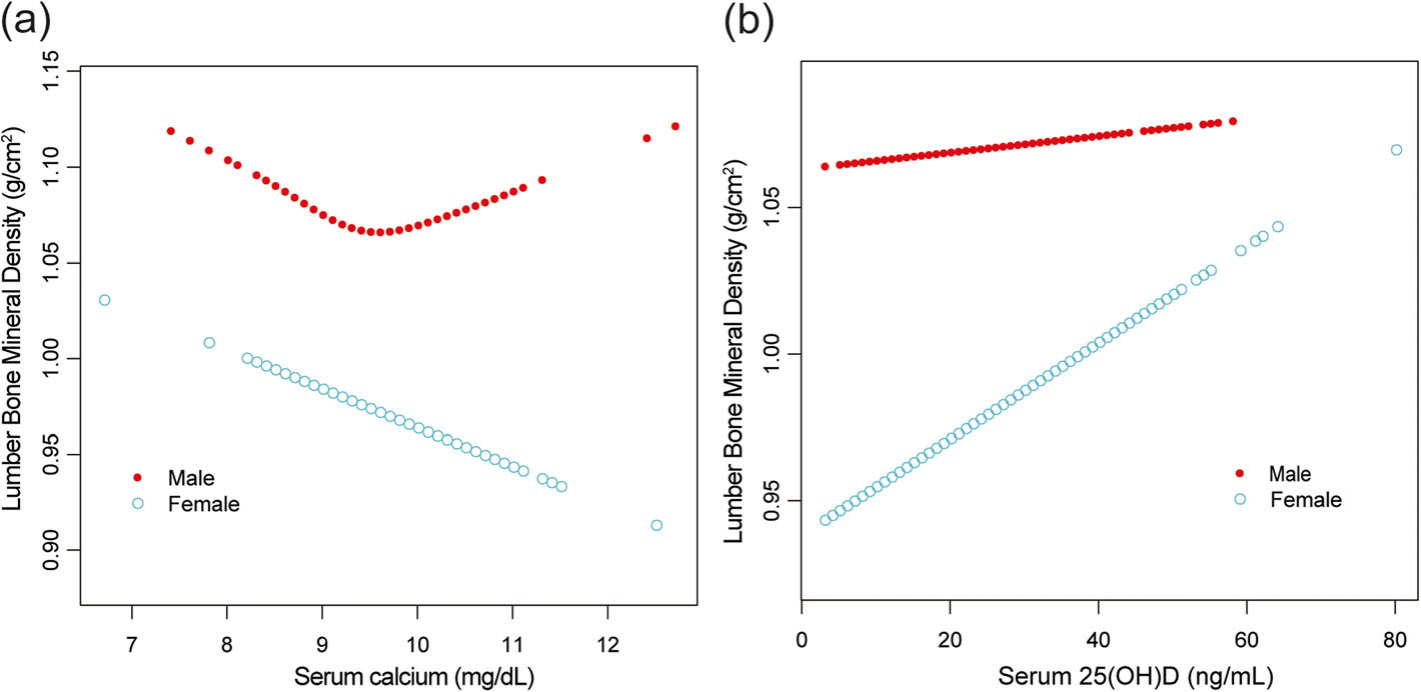
**a**, **b** The associations between serum calcium, serum 25(OH)D and lumbar bone mineral density stratified by sex. Age, race/ethnicity, body mass index, education, income to poverty ratio, physical activity, smoking behavior, and alcohol consumption were adjusted

**Fig. 5:**
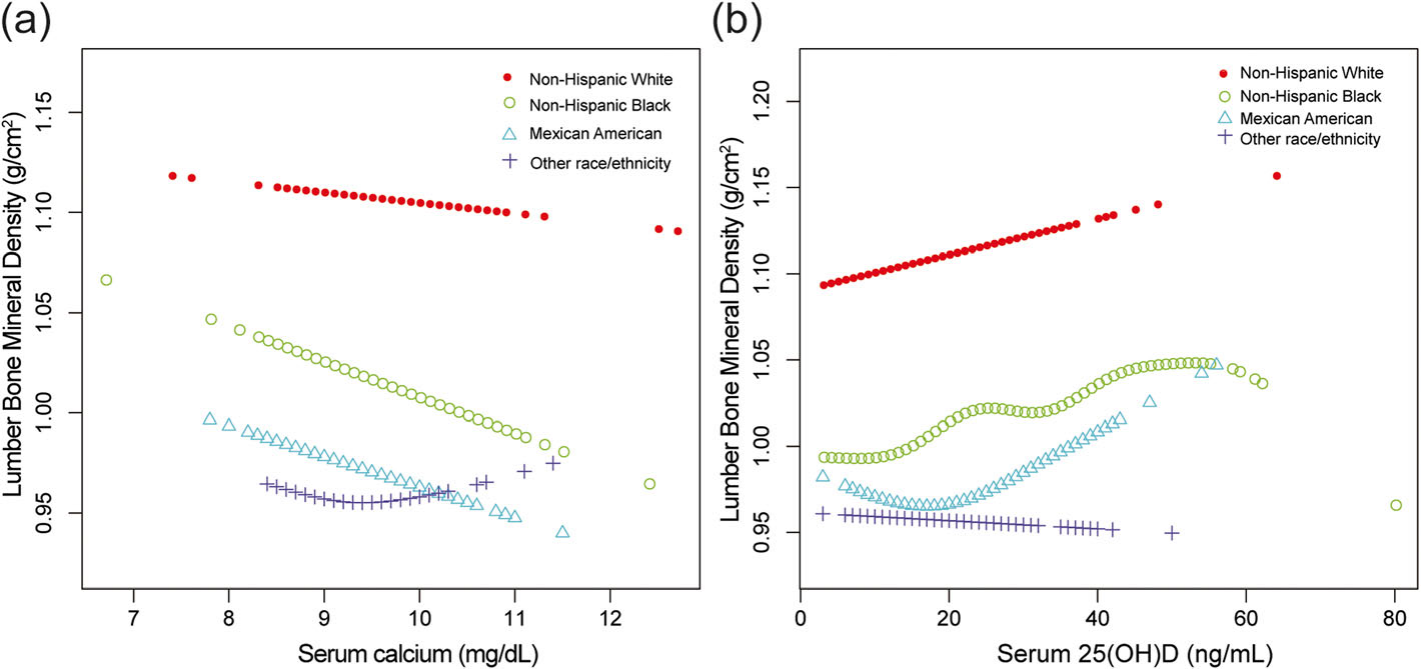
**a**, **b** The associations between serum calcium, serum 25(OH)D and lumbar bone mineral density stratified by race/ethnicity. Age, sex, body mass index, education, income to poverty ratio, physical activity, smoking behavior, and alcohol consumption were adjusted

In the subgroup analyses, we found that the association between serum 25(OH)D and lumbar BMD did not differ in different age groups, between men and women (Figs. 3 and 4), but this association followed a U-shaped curve in Mexican Americans (Fig. 5). Still, we examined the threshold effect using two-piecewise linear regression models (Table 3).

## Discussion

In this study, we used these representative samples of NHANES 2001–2006 to evaluate the associations between serum calcium, 25(OH)D and lumbar BMD in older adults. The results revealed a negative association between serum calcium and lumbar BMD, and a positive association between serum 25(OH)D and lumbar BMD. Specifically, the association between serum calcium and lumbar BMD in males followed a U-shaped curve. For males, lumbar BMD increased with serum calcium up to the turning point (9.6 mg/dL).

Despite the fact that calcium is the primary nutrient of interest in bone health, the information from previous studies linking serum calcium and BMD is limited. Recently, Cerani et al. [5] conducted a Mendelian randomization study, their data suggested that increased serum calcium levels did not increase BMD or provide clinically relevant protection against fracture. In a longitudinal cohort study consisting of 381 participants conducted by Dalemo et al. [6], the results revealed there was no correlation between baseline calcium and BMD at follow-up. Their data also showed patients with elevated serum calcium levels at baseline had osteoporosis more often than controls 10 years later. In the present study, we found a negative association between serum calcium and lumbar BMD. According to the STROBE statement [26], we use subgroup analysis to make better use of the data. In the subgroup analysis, this association was no longer significant among males and other race/ethnicity.

In the NHANES III study (1988–1994), Heike et al. [19] found the serum 25(OH)D positively correlated with the total hip BMD in older adults, and this association was strongest in whites. Some other cross-sectional studies supported this positive association of 25(OH)D with BMD [18–20]. Recently, Sun et al. [13] performed a Mendelian randomization study to investigate the association between vitamin D levels and total BMD, and their data showed increased vitamin D could not improve BMD in the general population. Our finding was consistent with the NHANES III study [19]. The strongest association between 25(OH)D and lumbar BMD was in whites, and this association followed a U-shaped curve in Mexican Americans. Therefore, the reasons for these different conclusions may be the heterogeneity among studies, including study size, study design, and differences in participant selection, such as age, sex, and race/ethnicity. Nevertheless, our results supported that increased 25(OH)D level would be beneficial to bone health in the population with 25(OH)D deficiency.

The representative samples of the multiracial population are included in this study to better generalize of the US population, and this large sample size allowed us to perform further subgroup analyses. This is the biggest strength of this study. There are some limitations. First, due to the nature of the cross-sectional study, we cannot determine whether higher vitamin D levels or lower serum calcium influence change in BMD over time, and the causality cannot be assessed. Second, we excluded participants with cancer or malignancy because these special populations have a great influence on serum calcium and BMD. Thus, the conclusions in this study cannot be used for them. Third, we did not adjust other variables. Therefore, the bias caused by other potential confounding factors is not excluded.

## Conclusions

In conclusion, our results suggested that serum calcium negatively correlated with lumbar BMD, and serum 25(OH)D positively correlated with lumbar BMD in older adults. However, the association between serum calcium and lumbar BMD in males followed a U-shaped curve.

## Abbreviations

BMD: Bone mineral density
BMI: Body mass index
NHANES: National Health and Nutrition Examination Survey
